# How changing quality management influenced PGME accreditation: a focus on decentralization and quality improvement

**DOI:** 10.1186/s12909-017-0937-9

**Published:** 2017-06-02

**Authors:** Nesibe Akdemir, Kiki M. J. M. H. Lombarts, Emma Paternotte, Bas Schreuder, Fedde Scheele

**Affiliations:** 1 Department of Medical Education, OLVG Teaching Hospital, P.O. Box 9243, 1006 AE Amsterdam, The Netherlands; 2 Professional Performance Research Group, Center for Evidence-Based Education, Academic Medical Center, University of Amsterdam, Amsterdam, The Netherlands; 3 Royal Dutch Medical Association, Specialist Physicians’ Registration Committee, Utrecht, The Netherlands; 4 School of Medical Sciences, VU Medical Center, Amsterdam, The Netherlands; 5 Athena Institute for Transdisciplinary Research, VU, Amsterdam, The Netherlands

**Keywords:** Accreditation, Medical education, Postgraduate medical education, Quality, Quality management, Quality improvement, Decentralization, Self-evaluation

## Abstract

**Background:**

Evaluating the quality of postgraduate medical education (PGME) programs through accreditation is common practice worldwide. Accreditation is shaped by educational quality and quality management. An appropriate accreditation design is important, as it may drive improvements in training. Moreover, accreditors determine whether a PGME program passes the assessment, which may have major consequences, such as starting, continuing or discontinuing PGME. However, there is limited evidence for the benefits of different choices in accreditation design. Therefore, this study aims to explain how changing views on educational quality and quality management have impacted the design of the PGME accreditation system in the Netherlands.

**Methods:**

To determine the historical development of the Dutch PGME accreditation system, we conducted a document analysis of accreditation documents spanning the past 50 years and a vision document outlining the future system. A template analysis technique was used to identify the main elements of the system.

**Results:**

Four themes in the Dutch PGME accreditation system were identified: (1) objectives of accreditation, (2) PGME quality domains, (3) quality management approaches and (4) actors’ responsibilities. Major shifts have taken place regarding decentralization, residency performance and physician practice outcomes, and quality improvement. Decentralization of the responsibilities of the accreditor was absent in 1966, but this has been slowly changing since 1999. In the future system, there will be nearly a maximum degree of decentralization. A focus on outcomes and quality improvement has been introduced in the current system. The number of formal documents striving for quality assurance has increased enormously over the past 50 years, which has led to increased bureaucracy. The future system needs to decrease the number of standards to focus on measurable outcomes and to strive for quality improvement.

**Conclusion:**

The challenge for accreditors is to find the right balance between trusting and controlling medical professionals. Their choices will be reflected in the accreditation design. The four themes could enhance international comparisons and encourage better choices in the design of accreditation systems.

## Background

Postgraduate medical education (PGME) programs are designed to provide high-quality training for residents [1–8]. Assessing the quality of medical education through accreditation is common practice worldwide [9–12]. Accreditation is intended to improve the quality of PGME [9, 13, 14]. Although accreditation is usually a top-down process with a considerable impact on PGME programs, critical literature concerning its design is scarce [15–17]. Understanding and appraising its functional components will enhance not only its design but also its effective use by both accreditors and teaching hospitals striving for excellence. This study aims to understand how changing views on educational quality and quality management have impacted the design of PGME accreditation in the Netherlands.

Whereas views on PGME influence the content of accreditation, an effective accreditation design will influence the quality of PGME. Over the past several decades, changing views on educational quality have resulted in the transformation of the content, structure and accreditation of PGME. Reforms of medical education occurred, for instance, after the publication of the Flexner Report in 1910 and the publication of the New Carnegie Foundation Report in 2010 [18]. In 1910, the emphasis was on the standardization of education. By 2010, this emphasis had shifted to a tailored learning process and standardization of outcomes, often with the use of competency frameworks [18]. Standards established a century ago might have lost their meaning in the current context. To understand current accreditation systems, it may be helpful to indulge in the historical course of their development.

How accreditation is conducted is shaped by changing views on quality management. Accreditation is a form of quality management, [19] which is defined as all activities designed to achieve and sustain high-quality output [6]. Quality assurance, quality improvement and quality control are examples of prominent approaches pursuing quality management [5, 20–22]. Quality assurance emphasizes the importance of complying with minimum standards, [23] whereas in quality improvement, the emphasis is on striving for excellence [8]. These two partially overlapping approaches are focused on the process of quality management. Quality control is focused on the output. For an overview of the different terms, please see the Definition box.

Accreditation may have consequences for what is being accredited. For example, accreditation claims to determine what educational ‘quality’ is and what it is not [9, 13, 14]. This means that if an institution does not comply with the standards, its PGME program may be discontinued by the accreditor [24–26]. This indicates the power exerted by the accreditor. Although this power is accepted and the importance of accreditation is recognized by program directors and residents, [27, 28] there is limited evidence for the benefits of different choices in its design [1, 14, 29, 30]. Benefits of different accreditation systems should be transparent and fit for critical appraisal [26, 31].

By outlining the underlying principles of the Dutch PGME accreditation system, we may pave the way towards a more theoretical discussion about the challenges and benefits of accreditation design in PGME. Therefore, our main question is the following: How have changing views on educational quality and quality management impacted the design of PGME accreditation in the Netherlands?

To answer this question, we choose an explorative qualitative approach and conduct a document analysis.

## Methods

This study explored the formal documents defining the Dutch PGME accreditation system spanning the past 50 years. These documents determine what was, what is and what will be considered to be quality. The changes over time in these documents will help to identify changes in educational quality and quality management.

### Postgraduate medical education in the Netherlands

In the Netherlands, there are 33 registered hospital-based medical specialties, each with their own specialty college. These colleges define their PGME programs. The duration of the specialty training varies between four and 6 years. Specialty-specific PGME training is regionally provided in a joint effort by an academic center in collaboration with multiple affiliated non-academic teaching hospitals. Residents are supervised by the program director and the team of clinical educators.

### The Dutch accreditation system for PGME

The Royal Dutch Medical Association (KNMG) is the professional authority and the regulator of PGME. The KNMG has two bodies concerned with the accreditation of PGME: a legislative college consisting of medical professionals and societal stakeholders (CGS) and an executive body consisting of medical professionals and legal advisors (RGS). The medical professionals are responsible for decision-making concerning accreditation, and the advisors are societal stakeholders and representatives of medical residents [32].

The CGS is responsible for setting the standards for accreditation for all specialties, while the RGS’ role is monitoring compliance with these standards. The accreditation cycle is 5 years. Participation in the accreditation process is mandatory for hospitals providing residency training.

In addition to the general standards for hospital-based specialties, there are specific standards for each PGME program. This document contains standards regarding the outline of the training program, for example. The third official document is the national curriculum of the medical specialty. This national curriculum is not expressed in standards, but is a description of the curriculum of the PGME programs. It is mainly formulated in aims, competences, learning opportunities and assessments.

### Study design

#### Selection of documents

We included the main reforms of the (1) general standards for hospital-based medical specialties since the foundation of the CGS in 1961. Non-hospital specialties such as general practice are regulated in separate documents and are therefore not included in this study. The (2) specific standards and (3) national curricula of internal medicine and general surgery were also included. The specific standards and the national curriculum of internal medicine and general surgery were used because they are both characteristic, large specialties with a relatively long history and are therefore the most informative. National curricula have existed since 2007, but we included only the most recent. The (4) preamble section and explanation section of the general standards and specific standards are useful as clarifications and were also included. The (5) annals of the meetings of the CGS before implementation of the new accreditation standards were gathered to explore the discussions occurring in the development of the general standards. The (6) policy documents of the Ministry of Health mentioned in the annals or explanation section of the general standards were also collected.

Currently, the accreditation standards are in a reform process, and the CGS has outlined the future accreditation system for 2018 in a formal vision document. This document outlines the future system and its main principles, and has been approved by the RGS. Therefore, we considered this (7) vision document relevant, and it was also included in the document analysis.

#### Data collection

All documents mentioned in numbers 1–5 above were retrieved via the archive collection of the Royal Dutch Medical Association (KNMG). From the annals of the CGS, only the paragraphs mentioning the reform of the general standards were used. The policy documents of the Ministry of Health mentioned in the annals of the CGS were retrieved online through the website of the Ministry [33]. The vision document of the 2018 accreditation system was obtained directly from the CGS secretary.

There were major reforms of the general standards in 1966, 1999 and 2004 that represent milestones in accreditation system design. Between these major reforms, there were also minor adjustments, which were not included in this study. We used the general standards of 1966, 1999, 2004 and the planned 2018 reform in this study. The most recent reform originated in 2004 and is still in effect, although it has been updated with minor changes that are included in this analysis.

The specific standards of internal medicine are from the years 1972, 2000 and 2011. The specific standards of general surgery are from the years 1969, 2000 and 2013. After reforms of the general standards, specialty colleges are obliged to update their specific standards.

The national curriculum was introduced in 2007. Because of this recent introduction, the national curriculum of internal medicine (2015) and the national curriculum of general surgery (2015) were included in the data from the 2004 system (2004–present).

#### Analysis

The document analysis was conducted using a template analysis technique [34]. The approaches of quality management (quality assurance, quality improvement and quality control) were used as a priori themes in the initial template. Quality management needs a context to acquire meaning, in our case, PGME. Therefore, we decided to combine the approaches of quality management with PGME quality domains. We used three PGME quality domains: quality of education (curriculum design: e.g., description of the structure of PGME programs), residency performance (intermediate outcomes during training: e.g., assessing the progress of the resident) and quality of physician practice (final outcome of training: e.g., entrustment decisions) [14]. Thus, quality assurance, quality improvement, quality control, quality of education, residency performance and quality of physician practice were the six a priori themes. From there, the template was developed through iteratively collecting and analyzing data.

As is common practice in discourse analysis, we included both primary (1, 2, 3 and 7) and secondary (4, 5, and 6) documents [35]. The primary documents are the most relevant texts for the research question, and the secondary documents are helpful to understand the phenomenon under study [35]. Therefore, only the primary documents were coded. The main researcher (NA) coded each reform of the general standards, specific standards, national curricula and the vision document of the future system with the a priori themes using the software package MAXQDA12. The themes were discussed by the research team to develop the final template. All the coded fragments were subdivided by the year of the reforms to map the shifts over time. A second researcher (EP) checked these coded fragments to verify the themes. The discrepancies between the two researchers were discussed, and this resulted in minor alterations of the final template. The final template was used to map the shifts over time regarding the specific themes.

## Results

All three past major reforms of the general standards (1966, 1999 and 2004) were induced by the Minister of Health. By contrast, the vision document of the future accreditation system was born from the intrinsic incentive of the medical profession, as stated: ‘*This reform was necessary because of innovation in PGME, recent developments in healthcare (e.g., integrated healthcare), and decentralization prevailing in quality policy.*’

### Template for PGME accreditation

From the formal policy documents published by the CGS (primary documents), two themes emerged. These were the objectives of accreditation and the actors’ responsibilities. The quality management approaches of the initial template were gathered under the heading of the quality management approach. The four themes comprising objectives of accreditation, PGME quality domains, quality management approach and actors’ responsibilities were the main elements of the system and are described below. The structure of the results was inspired by Sinek’s Golden Circle: why an organization exists and its core beliefs, how the organization fulfills its core beliefs, and what the organization does to fulfill its core beliefs [36].

First, the objectives of accreditation of PGME programs will be described: why accreditation? Subsequently, a focus on accreditation of PGME will be provided: what is accredited? Next, the instruments used for the objectives of accreditation will be explored: how are the objectives met? Finally, the responsibilities of the different actors in PGME accreditation will be discussed: who is involved? A summary of the themes and their intensity of use in the accreditation systems over the past 50 years is shown in Table 1.

**Table 1:** PGME accreditation structured according to Sinek’s Golden Circle

Accreditation			Former systems		Current system	Future system
			1966	1999	2004 - present	2018
Why	Primary objectives	*Quality of PGME*	+−	+	++	++
		*Quality of health care*	−	−	+−	+
	Secondary objectives	*Standardization*	++	++	++	+−
		*Coaching*	−	−	−	+−
		*Self-evaluation*	−	−	+−	++
		*Accountability*	−	+−	+	++
What	PGME quality domains	*Quality of education*	+	+	++	++
		*Residency performance*	−	−	+	++
		*Quality of physician practice*	−	−	+−	++
How	Quality management approach	*Quality assurance*	+−	+	++	+−
		*Quality improvement*	−	−	−	++
		*Quality control*	−	−	−	+
Who	Actors’ responsibilities	*Resident*	−	+−	++	++
		*Clinical educator(s)*	++	+	+	+
		*Organization*	−	+−	+−	++
		*Accreditor*	++	+	+	+−

This is an overview of overall shifts in the intensity of use of themes over a period of time based on qualitative data: ++ intensive, + frequent, + − moderate, − limited, −- never

## Why are PGME programs accredited?

### Primary objectives

We identified primary and secondary objectives in the accreditation system. The primary objectives were the quality of PGME and the quality of health care. Traditionally, the quality of PGME has been assessed separately from the quality of health care. However, in the current system, the quality of health care is given more weight in the assessment of the PGME programs. The national curricula have particularly contributed to the relationship between PGME and health care, e.g., by linking (generic) competences to clinical practice. In the proposed future system, the importance of the quality of health care is further emphasized through one of the basic principles of the system: ‘*The patient, client and other stakeholders all have an interest in qualified professionals who can provide the best care tailored to the individual needs of different stakeholders*.’

### Secondary objectives

The secondary objectives are ways to realize the primary objectives and were identified as: standardization, coaching, self-evaluation and accountability.

#### Standardization

Standardization has always been emphasized in the current and past accreditation systems. When standardization is prioritized, it means that it is important to comply with the standards set by the accreditor to be qualified for accreditation. However, a change of view was observed in the vision document of the future system concerning standardization in a quality improving perspective: ‘not ticking boxes, but improving quality’. In the future system, standardization will be further elaborated from a quality improvement perspective. There will be a reduced set of general standards that have to be met. In addition to these standards, teaching hospitals will have to develop self-arranged local quality management systems.

#### Coaching

The accreditor can take an active coaching role in the accreditation process to guide the PGME program. In the past, the coaching role of the accreditor was not dominant, but in the vision document of the future system, this has slightly changed. The accreditor may focus on specific themes in which to take a guiding role.

#### Self-evaluation

Self-evaluation differs from coaching because the position of the accreditor is more passive. The accreditor gives the teaching hospital the flexibility to arrange its local quality management. In the past, self-evaluation was absent. In the future system, there will be a strong emphasis on self-evaluation through local quality management systems to improve the quality of PGME programs.

#### Accountability

Finally, accountability is also an essential item, which has changed over time and which has become more important. The accreditor can justify the PGME programs regarding different parties: e.g., the medical profession, government and society. In 1966, accountability was prioritized less and was focused more on the medical profession and government. Currently, the emphasis is on accountability to society. This was also noticeable in the vision document of the future system. Public interests are prioritized, and it is thus important to have transparency in accountability.

## What is accredited in these PGME programs?

### PGME quality domains

#### Quality of education

Quality of education, residency performance and quality of physician practice are the three PGME quality domains that we expected to be covered by accreditation. The general and specific standards mainly cover the quality of education.

The accreditation standards show that the content of PGME has hardly changed over the past 50 years. The curriculum still consists of the same subjects (e.g., duration, knowledge, skills, assessment strategy, mandatory meetings and responsibilities for the execution of the program). However, the content has been elaborated in more detail, mainly in the specific standards and national curricula. The current standards describe more extensively what should be done, by whom, when and how often.

The major change in the quality of education over time is that the curriculum came to be provided by a team of clinical educators instead of one single educator. Thus, education became a team effort. Moreover, substantial adjustments were seen within the standards concerning some subjects, such as the duration of PGME. In previous systems, the duration of PGME was based on the medical specialty for which the resident was trained. In the current system, the duration of PGME is based on the performance of the resident with a specialty-dependent minimum.

The future system will still be based on a competency framework (CanMEDS competences), bolstered by current themes in society (e.g., elderly care, leadership skills and efficiency) and already established educational principles.

#### Residency performance and quality of physician practice

In the early days, the description of residency performance was limited, and the quality of physician practice was missing from the accreditation system. Since the introduction of the National Curriculum in 2007, residency performance and quality of physician practice have been described specifically for each specialty. The future system will have several (intermediate and final) outcome indicators, e.g., patient satisfaction related to residents and data from alumni. The vision document of the future system instructs specialty colleges to define (measurable) quality outcomes. These outcomes are important for the self-evaluation of the teaching hospital.

## How are PGME programs accredited?

### Quality management approach

The number of standards has increased enormously since the first reforms. In 1966, the CGS defined approximately 80 standards; in 1999, 120 standards were observed, and since 2004, the general standards counts approximately 290 standards. If we also include the specific standards and the national curriculum, the current accreditation system has expanded greatly. The number of standards will be reduced in the proposed future system. The general standards will consist of only a limited number of standards based on quality assurance supplemented with specialty-specific outcome indicators. With this input, the teaching hospital is allowed to arrange its own local quality management.

#### Quality assurance

The results showed that most standards have been based on a quality assurance approach. We noticed that nearly all standards in the general standards and specific standards are process-based. The method of evaluation seems to have remained steady over the past 50 years, as PGME has been evaluated through one major instrument, namely, the approach of what is called visitation or on-site visits, with peer review and self-assessment. The emphasis on quality assurance and the traditional visitation has always been very important for the assumed quality of PGME. The future system will be based on both assurance and improvement with an emphasis on the proportionality of the number of minimum standards. As the vision document of the future system indicates: *‘The search is for an accreditation system based on clear standards in which the professional has plenty of room for personal interpretation. ‘.*

#### Quality improvement

The former systems did not include standards containing quality improvement. The occurrence of quality improvement standards in the current system is still sporadic; e.g., *‘The program committee is a consultative body that maintains and promotes a convenient and safe clinical learning climate and has therefore the following tasks: (…) monitoring and promoting the quality of the PGME programs available in the teaching hospital.’* The emphasis on quality improvement will increase in the future system. This aim will be primarily carried out using encouraging and supervising local quality management.

#### Quality control

The only outcome-based standard that has existed since 1966 is the following: *‘(...) after finalizing the program to practice independently and accurately the medical specialty for which he is trained for.’* A new outcome-based standard was added to the general standards in the 2004 system. The new standard inspired by CanMEDS describes the generic competences in outcome-based standards. Since the early 1990s, outcome-based standards have been desired, but all the attempts failed to increase outcome-based standards until the introduction of the national curriculum in 2007. This was the first endeavor to define the expected outcomes during and at the end of residency training. The aim is still to have more outcome-based standards in the future system. The specialty colleges are responsible for developing these specific outcomes and other quality requirements. In the future system, the method of evaluation will change significantly. Until now, the emphasis has been on quality assurance, and this will shift to quality control by means of outcome indicators in the future system. Furthermore, the accreditor will be able to visit to examine specific themes, e.g., innovations in health or current themes in society.

## Who is involved in accreditation?

### Actors’ responsibilities

The actors’ responsibilities disclosed in the accreditation standards describe the role of each actor at different levels in PGME. These actors consist of residents, clinical educators, organizations and accreditors. The number of actors in residency education has increased with time. According to the vision document of the future system, the number of actors will further increase with the addition of two new actors in accreditation: regional boards, which are eight boards responsible for medical education in the eight regions in the Netherlands, and the specialty colleges. The vision document does not detail the distinct responsibilities of all actors, but these responsibilities will be further elaborated in the future.

#### Resident

In 1966, the resident was not an active actor. The tasks of the resident were placed under the responsibility of the clinical teacher(s). In 1999, this changed slightly, and the resident gained more responsibilities. In 1999, the responsibilities of the resident were discussed in the CGS, and the members agreed that ‘*the resident cannot hide behind his clinical educator*’. The current role of the resident is more extensive than before. This means that the resident is responsible for his own training and also for the care he provides. This is also emphasized in the National Curriculum, where the focus lies on both the residents and the clinical educators. In the future, the resident’s role will remain important.

#### Clinical educators

In 1966, the responsibility of training residents belonged to a single clinical educator instead of a team of clinical educators. The team of clinical educators with a program director as a formal head of the program was introduced after 1999. The program director shared his responsibilities with the team. In the current system, the emphasis on the team’s responsibility is further increased, although the ultimate responsibility for residency training still falls on the program director. This team responsibility will be maintained in the future system.

#### Organization

Since 1999, program directors have shared responsibilities with the team of clinical educators, as well as with program directors of other specialties in the same teaching hospital. This is called a program committee, and it is compulsory for each teaching hospital. The program committee’s tasks include:

‘(…) *maintaining and promoting a supportive and safe clinical learning climate* (…)’.

and

‘*monitoring and stimulating the quality of PGME programs in the teaching hospital*.’

In 1966, there was no program committee. In the future system, the program committee will have a key role in the local quality management and the responsibility for quality improvement of PGME.

#### Accreditor

In 1966, the evaluation of PGME was structured centrally. The accreditor was responsible for the quality of PGME. Since 1999, this responsibility has slightly shifted to a more local level (program committee). The program committee became an important body for the delegation of accreditor responsibilities regarding the quality of PGME. The shift of responsibilities to a more local level represents decentralization.

The responsibilities of the accreditor will change further in the future system. Accreditation will focus on the local quality management of the teaching hospital. Depending on the excellence of the local quality management, the accreditor can decide how strictly a teaching hospital should be evaluated. As a result, good local quality management will be rewarded with more trust given to the teaching hospital and less intensive monitoring by the accreditor.

## Discussion

This exploratory study investigated the changing views on educational quality and quality management of PGME. Analysis of 50 years of accreditation documents helped to identify shifts in four themes: objectives of accreditation, PGME quality domains, quality management approaches, and actors’ responsibilities. These themes may be useful for identifying and comparing choices within accreditation design for PGME. We found an increased emphasis on quality improvement of PGME programs, which will likely continue in the future. The current accreditor intends to stimulate this with an increased focus on self-evaluation and decentralization. Defining outcomes for residency performance and quality of physician practice could further improve local quality management.

Our findings resonate with studies conducted in health care regulation using responsive regulatory theory (RRT), providing recommendations that are aligned with the Dutch future planning. Responsive regulation consists of mechanisms that are responsive to the context, conduct and culture of those being regulated [37]. Responsive regulation originates as a theory of business regulation and is now also used for a wide range of public governance applications [7].

In RRT it is essential to stay focused on the goals, [38] which ideally must determine the choices made in the accreditation design. Still, it could be that how the accreditation body strives for its core beliefs may not be entirely in line with its goals.

For example, in our study, we noticed an increased number of standards due to detailing the initially generally expressed standards. The expansion of strict standards implies that there is less room for teaching hospitals to shape their own program, which contrasts with the current shift towards decentralization and self-evaluation. After all, decentralization and self-evaluation accompany trust in the teaching hospital. This could also benefit the intrinsic motivation of teaching hospitals, as it is already known that external enforcement or rewards may undermine intrinsic motivation, [39–41] in particular, when they are perceived as intrusive or not supportive [39].

Moreover, research in health care services shows that the higher the number of standards, the less likely all standards will be audited [42]. The increase in standards and the aimed reduction, on the one hand, and the rise of self-evaluation and decentralization of incentives for improvement, on the other hand, also suggest that the accreditor must search for the right balance between controlling and trusting the teaching hospitals [43].

The aim of the Dutch accreditor is to reduce the number of standards in the future system and to create more flexibility for teaching hospitals. However, self-regulation does not guarantee less rigid approaches or paperwork. Multiple studies in health care regulation show that self-regulation led to the opposite: more rules and standards [42, 44].

Quality management and educational quality are intertwined. Because of quality management’s current strive for the decentralization of quality improvement, professionals are faced with new responsibilities and challenges regarding educational quality. This provides new opportunities for professionals to improve educational quality and could accelerate a change in views on educational quality. In an ideal situation, this could result in a bottom-up movement towards outcome-based standards for accreditation.

### Strengths and limitations

To the best of our knowledge, this is the first study that attempts to structure the strategic perspectives of accreditation systems for PGME based on the history of an accreditation system. The resulting framework helps to evaluate accreditation systems worldwide. Systems can be mapped and compared with the same elements. This can contribute to an international discussion of the effectiveness of distinct elements.

This study is based on formal documents that give an overall picture of PGME accreditation. We should stress that there are operational levels that are not examined in this study. To better understand the effects of accreditation systems, it is necessary to study the process in practice as well as in a document analysis.

### Suggestions for future research

This study provides insight into the development of an accreditation system. The framework described could contribute to the study and appraisal of accreditation systems worldwide. The framework described needs further development because this study analyzed only development over time in the Netherlands. There is also a need for further exploration of different jurisdictions to analyze different contexts [23, 45]. This may change or improve our framework of accreditation. Moreover, an international perspective on accreditation systems is part of a desperately needed search for best practices.

## Conclusion

This study has outlined the development of the PGME accreditation system in the Netherlands over the past 50 years. The current system is facing new challenges, and the focus on decentralization and quality improvement brings new strains. Historically, the system was structured centrally and followed a top-down process. The focus on decentralization in the Dutch situation gives the teaching hospitals flexibility and responsibility to tailor their quality management. If teaching hospitals adapt to this new responsibility, this change could result in a more user-driven system instead of a top-down bureaucratic system. An ideal situation in which teaching hospitals are intrinsically motivated to strive for excellence may lead to bigger improvement steps. If carried out as intended, this form of decentralization will result in cooperation with and trust in the professionals, instead of deterrence and control by the accreditor.

Trust and discretion, on the one hand, and distrust, enforcement and deterrence, on the other hand, are opposite sides of a coin. In the past 50 years, accreditors have searched for the right balance between these two poles. This exercise is an extremely delicate one, and we must focus on it in future research. The question is which of the two will outweigh the other, for this will be reflected in the design of accreditation systems.

## Abbreviations

CGS: Dutch Legislative College for Accreditation of Postgraduate Medical Education
KNMG: Royal Dutch Medical Association
PGME: Postgraduate Medical Education
RGS: Specialist Physicians’ Registration Committee
